# AGING MARKERS IN CONTEXT OF MORBIDITY, FRAILTY, AND GERIATRIC ASSESSMENTS: LONGITUDINAL RESULTS FROM BASE-II

**DOI:** 10.1093/geroni/igae098.2619

**Published:** 2024-12-31

**Authors:** Valentin Vetter, Johanna Drewelies, Jan Homann, Sandra Düzel, Simone Kühn, Lars Bertram, Denis Gerstorf, Ilja Demuth

**Affiliations:** Charité - Universitätsmedizin Berlin, Berlin, Berlin, Germany; Max Planck Institute for Human Development, Berlin, Berlin, Germany; University of Münster, Münster, Nordrhein-Westfalen, Germany; Charité - Universitätsmedizin Berlin, Berlin, Berlin, Germany; Max Planck Institute for Human Development, Berlin, Berlin, Germany; University of Lübeck, Lübeck, Schleswig-Holstein, Germany; Humboldt University Berlin, Berlin, Berlin, Germany; Charité - Universitätsmedizin Berlin, Berlin, Berlin, Germany

## Abstract

Several markers are available that aim at the quantification of inter-individual differences in biological age. While many of these markers have been evaluated individually, there are few comparisons in terms of their association with age-related outcomes within the same dataset available. In this study, we compare 13 different aging markers in relation to morbidity, frailty and geriatric assessments in 1,671 participants of the Berlin Aging Study II (BASE-II). Participants were on average 68.8 years old at baseline (SD = 3.7 years) and 51.6% of the participants were women. A follow-up examination of 1,083 participants was conducted on average 7.4 years later. Biological age was assessed as epigenetic age using DNA methylation (7-CpG, Horvath, Hannum, PhenoAge, GrimAge, DunedinPACE), telomere length, BioAge, BrainAge, SkinAge, Subjective Age, Subjective Life Expectancy, and Subjective Health Horizon. Aging markers were analyzed in the context of morbidity (overall morbidity, diabetes and diabetes associated complications, cardiovascular risk scores), frailty (Fried), Tinetti Mobility Test, falls in past 12 months, finger-floor distance, mini mental state examination, Center for Epidemiologic Studies-Depression scale, activities of daily living (ADL), and mini nutritional assessment (MNA). Associations between ageing markers and outcomes were assessed with regression analyses adjusted for known confounders (age, sex, smoking, BMI). Preliminary results revealed strengths and limitations of the biomarkers analyzed with respect to the different domains of aging represented by the investigated age-associated outcomes. Cross-sectional and longitudinal results will be presented at the meeting and discussed in the context of the literature.

